# Curcumin ameliorates fatty liver hemorrhagic syndrome in broiler breeders by regulating lipid metabolism via the gut-liver axis

**DOI:** 10.1016/j.psj.2026.107650

**Published:** 2026-08-18

**Authors:** Jinling Ye, Yujie Huang, Ahmed Mohamed Fouad, Sai Zhang, Hebat Allah Kasem El-Senousey, Zhongyong Gou, Chang Zhang, Zongyong Jiang, Shouqun Jiang, Dong Ruan

**Affiliations:** a Institute of Animal Science, Guangdong Academy of Agricultural Sciences, State Key Laboratory of Swine and Poultry Breeding Industry, Key Laboratory of Animal Nutrition and Feed Science in South China, Ministry of Agriculture and Rural Affairs, Guangdong Key Laboratory of Animal Breeding and Nutrition, Guangzhou Guangdong, 510640, China; b Department of Animal Production, Faculty of Agriculture, Cairo University, Giza 12613, Egypt

**Keywords:** Fatty liver hemorrhage syndrome, Curcumin, Broiler breeder hens, Lipid metabolism, Gut microbiota

## Abstract

Fatty liver syndrome (FLS) is closely linked to disruptions in lipid metabolism, imbalances in the gut microbiome, and alterations in bile acid composition. Curcumin (CUR), a bioactive compound derived from turmeric, has been reported to exert anti‑inflammatory and hepatoprotective effects. The present study aimed to evaluate the potential of dietary CUR to mitigate fatty liver‑related metabolic disturbances in broiler breeder hens fed a low‑protein high‑energy (LPHE) diet. A total of 600 Qingyuan partridge broiler breeder hens (42 weeks of age) were used in a randomized experimental design. Birds were allocated to five dietary regimens, including a control diet (CON), a low‑protein high‑energy diet (LPHE), and LPHE diets supplemented with CUR at 100, 200, or 400 mg/kg. Each group consisted of six replicates with 20 birds per replicate. The feeding trial was conducted over an eight‑week period. The results showed that dietary supplementation with CUR at 100 and 200 mg/kg improved productive performance by reducing feed-to-egg ratio, abnormal egg rate, fat pad mass and lipid droplet in hepatic tissue versus hens fed the LPHE diet. In addition, CUR supplementation reduced plasma and hepatic levels of TG, TCHO, and LDL, as well as ALT activity and concentrations of TGF-β and TNF-α, while elevating IL-10 and IL-22 levels (*P*<0.05). Bile acid profiling demonstrated that DCA levels were increased in the LPHE_CUR200 group, while GCDCA, GDCA, and TLCA were reduced relative to LPHE group. Moreover, CUR corrected LPHE-induced disruptions in lipid metabolism and bile acid regulatory genes, including *FXR, BSEP, PPARα, CPT1α* and *CYP7A1*. Metabolomics demonstrated that LPHE altered hepatic metabolism, and it was partially restored in that of LPHE_CUR100 group. Dietary supplementation with 100 to 200 mg/kg CUR enriched *Lactobacillus* and *Peptococcus* populations in cecum. In summary, dietary inclusion of CUR at 100 to 200 mg/kg improved hepatic function, lipid metabolism in broiler breeder hens subjected to the LPHE diet, which may be regulated through the gut microbiota-Bile acid axis.

## Introduction

Fatty liver hemorrhagic syndrome (FLS) refers to a hepatic disorder featuring lipid processing imbalance caused by reduced fat breakdown and oxidation (Shini et al., 2020). Although FLS is not a disease essentially, but leads economic losses due to diminish ovarian follicles development, egg yield, and impaired feed conversion ratio as well as increasing mortality rate, as well as a higher proportion of substandard breeding eggs (Li et al., 2024; Yaqoob et al., 2024; Wang et al., 2025). FLS was associated with elevated levels of triglycerides (TG), total cholesterol (TCHO), low-density lipoprotein cholesterol (LDL) in blood and liver (Liu et al., 2025; Sun et al., 2025). Therefore, strategies to mitigate FLS have gained significant attention in recent poultry industry research..

Dietary imbalance, particularly a low-protein, high-energy (LPHE) feeding strategy, is frequently utilized to simulate FLS in laying hen models (Rozenboim et al., 2016; Zhang et al., 2024; Li et al., 2025). Plant-derived bioactive compounds as dietary supplements have attracted growing interest, as they may help reduce FLS in avian species. Curcumin (CUR), a *Curcuma longa*-derived polyphenol, has been extensively researched for its anti-inflammatory, antioxidant, and antimicrobial properties (Jabczyk et al., 2021; Khoshakhlagh et al., 2025; Jabczyk et al., 2021). It is worth noting that CUR modulates glycometabolism and lipid metabolism (Jabczyk et al., 2021; Matumba et al., 2024; Xie et al., 2019), boosts antioxidant capacity, suppresses lipid peroxidation and inflammatory cytokines production, and improves liver function (Kibitlewska et al., 2026).

Accumulating evidence suggests that gut microbiota-derived metabolites, including microbe-modified bile acids (BAs), contribute to the control of host lipid homeostasis by facilitating dietary lipid processing (Kaliannan et al., 2018; Mayneris-Perxachs et al., 2021; Li et al., 2022). Farnesoid X receptor (FXR), a bile acid-activated nuclear receptor, serves as a key mediator of intracellular bile acid signaling pathways. FXR activation plays a vital role in preserving bile acid homeostasis and contributes to the regulation of hepatic lipid metabolism and inflammatory processes (Zhang et al., 2024). CUR exerts its effects by regulating the BA-FXR pathway and inhibiting inflammation, as demonstrated in our previous research (Ruan et al., 2022). Although many potential mechanisms have been proposed, the exact mechanism by which CUR alleviates hepatic steatosis in broiler breeder hens is still not fully determined. Therefore, this study examined whether dietary CUR supplementation alleviated LPHE diet-induced FLS in broiler breeder hens through the gut microbiota-BAs axis.

## Materials and methods

Experimental animal procedures were executed in strict adherence to the Ministry of Science and Technology's guidelines for animal experimentation. Ethical authorization was granted by the Animal Care Committee of the Institute of Animal Science, Guangdong Academy of Agricultural Sciences, Guangzhou, People's Republic of China (Approval No: GAASISA-2024-028).

### Animals, diets and experimental design

Qingyuan partridge broiler breeder hens were selected for this experiment based on age uniformity and health condition. In total, 600 broiler breeder hens aged 42 weeks were obtained from Zhaoqing Yinongxing Breeding Development Co., Ltd. (Zhaoqing, China). In this experiment, five dietary treatments were evaluated using randomly assigned breeder hens. Each treatment included six replicates (20 birds per replicate), and birds were housed at two per cage. Five dietary treatments were applied in this study. control‑treated birds consumed a basal diet (CON), while those in the remaining groups were fed a low‑protein, high‑energy diet (LPHE) either without supplementation or supplemented with CUR at levels of 100, 200, or 400 mg/kg (LPHE_CUR100, LPHE_CUR200, and LPHE_CUR400). The experimental period extended over a total of eight weeks.

All diets used in the present study were prepared using a corn–soybean meal base. The basal diet's nutrient composition was designed based on national feeding standards (NY/T 3645-2020) from the Guangdong Academy of Agricultural Sciences' Animal Science Institute to align with recommended nutritional specifications for yellow-feathered chickens. Study-specific diets were prepared, and detailed feed composition and nutritional profile are shown in Table S1. The CUR was kindly provided by Guangzhou Cohoo Biotechnology Co., Ltd., (Guangdong, China; the purity > 98%).

Feed consumption was recorded daily at the replicate level throughout the trial. Egg production was assessed by documenting the daily number of total eggs, including broken and shell-less eggs, for each replicate group. In addition, all eggs produced were weighed individually and classified accordingly. At each replicate, egg production rate, mean egg weight, egg mass, and feed-to-egg ratio (feed efficiency) were evaluated as performance indicators. The data obtained were subsequently pooled for statistical evaluation.

### Biological sample processing and analysis

After the feeding trial, two birds per replicate closest to the average weight were anesthetized with isoflurane and sacrificed. Blood (approximately 5 mL) was collected from the wing vein into anticoagulant tubes and centrifuged at 3,000 × g for 10 min at 20-25°C to obtain plasma, which was stored at -80°C until analysis.

After blood sampling, liver and peritoneal fat were isolated, and organ weights were normalized to body weight and expressed as relative percentages. Three liver samples (approximately 100 mg each) from the mid-lobular region were collected for molecular and biochemical analyses. A portion of liver tissue was fixed in 4% paraformaldehyde for 48 h for histopathological assessment. Sections were stained with hematoxylin-eosin (H&E) and Oil Red *O*, and imaged using the PANNORAMIC SCAN II system (3DHISTECH, Budapest, Hungary). Remaining liver samples were stored at ultra-low temperature for bile acid determination and metabolomic profiling. Cecal samples were gathered immediately following sacrifice and preserved in a -80°C freezer until analysis.

### *Biochemical and enzyme-linked immunosorbent assa*y

Biochemical and immunological analyses were performed using plasma and liver samples to assess lipid profiles and hepatic enzyme activities. Nanjing Jiancheng Institute of Bioengineering supplied the commercial colorimetric assay kits used to determine plasma total cholesterol (TCHO), low-density lipoprotein (LDL), and high-density lipoprotein (HDL) concentrations, together with the activities of aspartate aminotransferase (AST) and alanine aminotransferase (ALT). The analyzed cytokines included interleukin-1β (IL-1β), interleukin-10 (IL-10), interleukin-22 (IL-22), tumor necrosis factor-α (TNF-α), transforming growth factor-β (TGF-β), and interferon-γ (IFN-γ). All immunoassays were carried out following standardized procedures supplied by the manufacturer (Jiangsu Meimian Industrial Co., Ltd., Zhangjiagang, China).

### Quantitative real-time PCR

Frozen liver tissues were homogenized for RNA extraction, followed by quantitative PCR analysis. Complementary DNA was synthesized and subjected to amplification following established protocols described previously (Ye et al., 2022). Oligonucleotide primers for the target genes were fabricated by Sangon Biotech Co., Ltd (Guangzhou, China), and detailed information is provided in Supplementary Table S2. Gene expression levels were calculated by the 2^^−ΔΔCt^ method. *β-actin* was chosen as the endogenous reference gene, and relative mRNA abundance was expressed in relation to the control treatment.

### Bacterial DNA isolation and 16S rRNA gene amplification

Microbial genome DNA was isolated from frozen cecal contents using a commercial kit, and the bacterial 16S rRNA V3-V4 hypervariable region was amplified with barcoded primers (27F and 1492R) for sequencing. PCR amplification was carried out using high-fidelity polymerase reagents, purified with a Qiagen gel extraction kit, and sequenced on an Illumina MiSeq platform by Majorbio Bio-Pharm Technology Co., Ltd. Taxonomic composition variations between treatment groups were evaluated using LEfSe. The Kruskal-Wallis test (H test) was applied to identify microbial taxa exhibiting significant differences between groups, following previously described analytical procedures (Ruan et al., 2022).

### GC-MS based metabolomics study

Samples using seven biological replicates per treatment group. For each sample, 50 mg of powdered liver tissue was extracted following previously described procedures (Xu et al., 2023). Raw GC–MS data processing, including peak detection, alignment, deconvolution, and integration, was carried out according to the established analytical workflow.

Multivariate statistical analyses were conducted after normalization to an internal standard. Unsupervised PCA and supervised OPLS-DA analyses were carried out using SIMCA-P v13 software (Umetrics, Umeå, Sweden) to examine metabolic differences among treatment groups. Differential metabolites were determined based on VIP values greater than 0.5 and absolute correlation coefficients |p(corr)| exceeding 0.5.

### Untargeted and targeted analysis of hepatic bile acids by UPLC–MS/MS

Untargeted metabolomic investigation of liver homogenate bile acids was adopted utilizing the UPLC–MS/MS platform. In addition, targeted profiling of hepatic bile acids was carried out based on a previously reported LC‑MS/MS method with minor methodological adjustments (Li et al., 2025).

### Statistical analysis

Pairwise comparisons between the control group and LPHE group were carried out with t-tests. One-way ANOVA was employed to statistically test differences among treatment groups. If significant differences were observed, the Duncan multiple comparison method was used to further identify specific intergroup differences. The statistical significance level was set at P < 0.05, and all statistical analyses were performed using SAS 9.4 (SAS Inst. Inc., Cary, NC).

Variations in gut microbial communities across experimental groups were explored using principal coordinate analysis (PCoA) implemented through the mixOmics framework. Relationships among intestinal metabolites, microbial composition, cytokine concentrations, and gene expression patterns were further assessed by Pearson’s correlation analysis, with statistical significance defined at *P* < 0.05.

## Results

### Laying performance

The data presented in Table 1 delineate the effects of experimental feeding regimens on laying productivity and adipose tissue parameters. Hens fed the LPHE diet exhibited lower laying rate and egg mass compared with those in the CON group, accompanied by an increase in feed‑to‑egg ratio, abnormal egg rate, and abdominal fat percentage (*P* < 0.05). Compared with the LPHE group, dietary supplementation with CUR at 200 and 400 mg/kg increased average egg weight and reduced liver index and abdominal fat percentage (*P* < 0.05). Moreover, supplementation with 100 or 200 mg/kg CUR significantly improved feed efficiency, reducing feed-to-egg ratio and abnormal egg rate (*P* < 0.05).

**Table 1:** Effects of low protein high energy diet supplemented with CUR on the egg laying performance of broiler breeder hens.

Indices	Treatments^1^					SEM	*P*-Value
	CON	LPHE	LPHE_CUR100	LPHE_CUR200	LPHE_CUR400		
Laying rate/%	57.75^A^	50.27^B^	53.39	51.66	49.01	1.464	0.062
Average egg weight/g	51.20	50.87^b^	51.17^b^	52.18^a^	52.38^a^	0.281	0.042
Egg mass/(g/d)	29.49^A^	26.26^B^	27.1	26.91	25.43	0.739	0.656
Feed to egg ratio/%	3.39^B^	4.14^A^^a^	3.95^b^	3.96^b^	4.29^a^	0.138	0.048
Broken egg rate/%	0.21	0.23	0.36	0.33	0.52	0.122	0.39
Abnormal egg rate/%	0.27^B^	0.55^A^^a^	0.48^b^	0.47^b^	0.52^a^^,^^b^	0.008	0.028
liver index/%	2.52	2.33^a^	2.29^a^^,^^b^	2.13^b^	2.10^b^	0.012	0.035
Abdominal fat percentage/ %	3.72^B^^b^	5.97^A^^a^	4.22^b^	4.26^b^	4.57^b^	0.004	0.003

Mean values within a row with no common superscript differ significantly (*P*<0.05). CON, broiler breeder hens were fed the basal diet; LPHE, broiler breeder hens were fed low protein and high energy diet; LPHE_CUR100, broiler breeder hens were fed low protein and high energy diet supplemented with 100 mg/kg curcumin; LPHE_CUR200, broiler breeder hens were fed low protein and high energy diet supplemented with 200 mg/kg curcumin; LPHE_CUR400, broiler breeder hens were fed low protein and high energy diet supplemented with 400 mg/kg curcumin.

a Capital letters indicate statistically significant (*P*<0.05) differences between control group and LPHE group by Student’s t-test; small letters indicate statistically significant (*P* < 0.05) differences of four groups (LPHE, LPHE-CUR100, LPHE-CUR200, LPHE-CUR400). Mean values within a row with no common superscript differ significantly (*P*<0.05).

b The *P*-value are representing the ANOVA analysis of four groups (LPHE, LPHE-CUR100, LPHE-CUR200, LPHE-CUR400).

### Hepatic histomorphological analysis

**Fig. 1:**
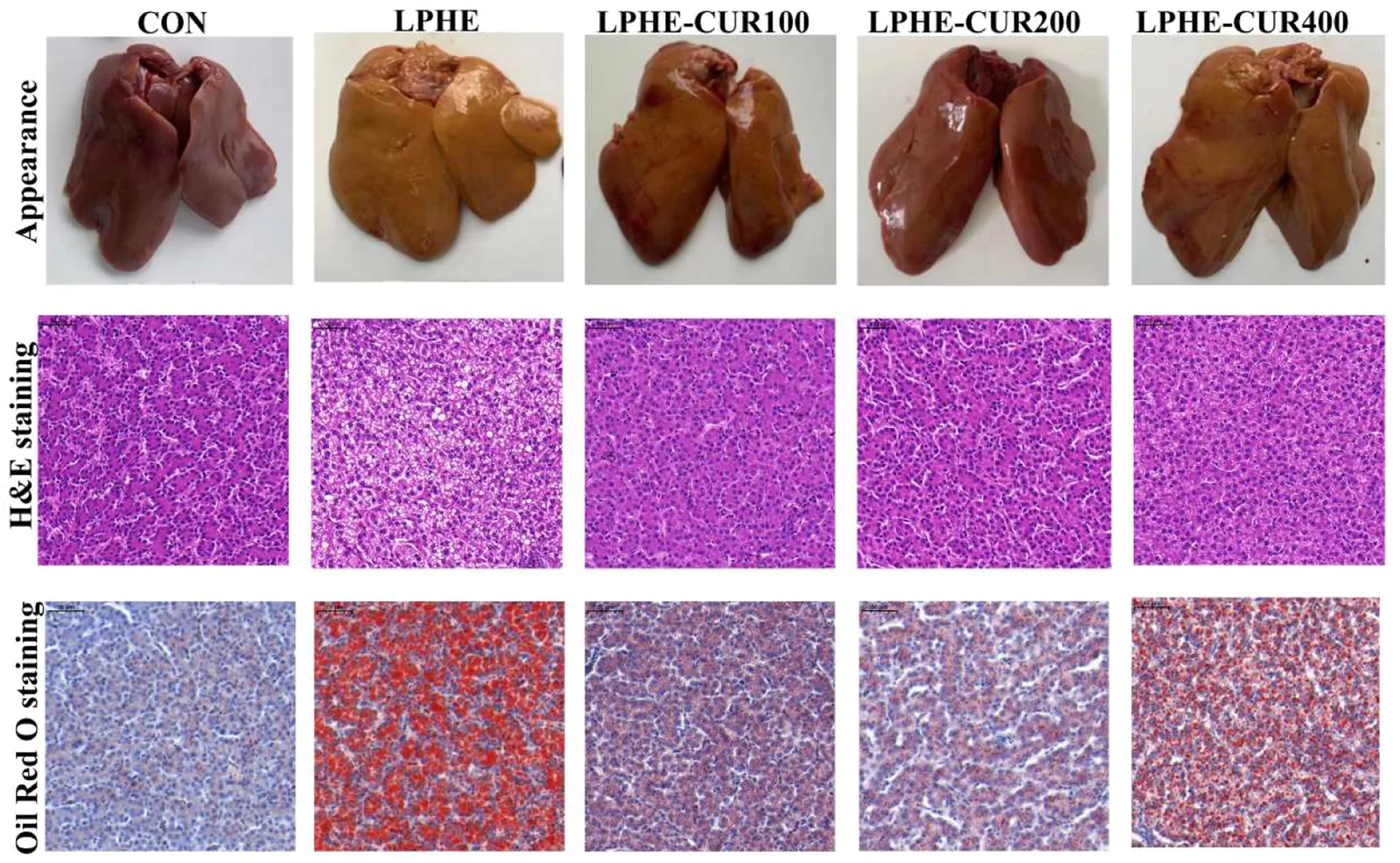
The effects of low protein with high energy diet supplemented with curcumin (CUR) on the pathological changes of the liver in broiler breeder hens. CON, basal diet; LPHE, low protein high energy diet; LPHE_CUR100, low protein high energy diet supplemented with 100 mg/kg curcumin; LPHE_CUR200, low protein high energy diet supplemented with 200 mg/kg curcumin; LPHE_CUR400, low protein high energy diet supplemented with 400 mg/kg curcumin.

As shown in Fig. 1, H&E staining and Oil Red *O* staining of liver tissues showed that the liver in LPHE group showed obvious enlargement, blunt and round edges, soft and fragile, appearing earthy yellow oil with a greater number of lipid droplets, compared with CON group. The fatty morphology of the liver in LPHE_CUR100, LPHE_CUR200 group was alleviated the above symptoms, with the most effective relief in LPHE_CUR200 group.

### The hepatic concentrations of biochemical indices and inflammatory cytokines

**Fig. 2:**
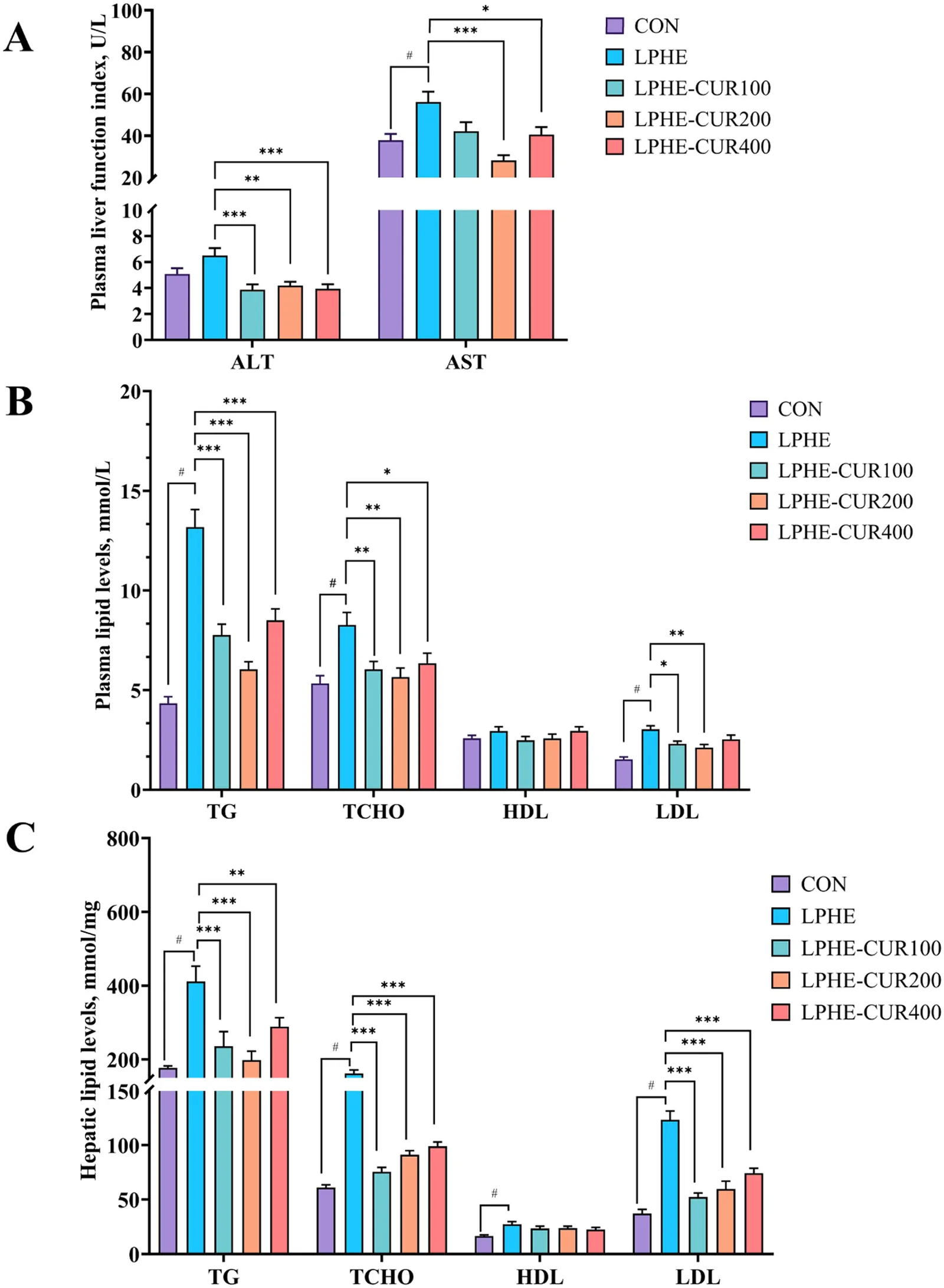
The effects of low protein with high energy diet supplemented with curcumin (CUR) on plasma biochemical indexes and hepatic lipid levels in broiler breeder hens (n=12). The concentrations of biochemical indexes in plasma (A-B) and hepatic lipid metabolism changes (C) were observed by biochemical reagent kits. CON, basal diet; LPHE, low protein high energy diet; LPHE_CUR100, low protein high energy diet supplemented with 100 mg/kg curcumin; LPHE_CUR200, low protein high energy diet supplemented with 200 mg/kg curcumin; LPHE_CUR400, low protein high energy diet supplemented with 400 mg/kg curcumin; Data are presented as mean value ± SD. # means the significant difference of LPHE compared to CON group, and # means P < 0.05. * means the significant difference of LPHE_CUR100, LPHE_CUR200, and LPHE_CUR200 compared to LPHE group, and * means P < 0.05, ** means P < 0.01, *** means P < 0.001.

As exhibited in Fig. 2, compared with the CON group, the LPHE group the LPHE group showed a significant increase in the plasma activities of AST (Fig. 2A) and the concentrations of TG, TCHO, LDL (Fig. 2B) (*P*<0.05). Compared with the LPHE group, the activities of ALT (Fig. 2A) and concentrations of TG, TCHO, LDL in plasma (Fig. 2B) decreased in the LPHE_CUR100 and LPHE_CUR200 groups (*P*<0.05). Meanwhile, the hepatic concentrations of TG, TCHO and LDL significantly decreased in CUR‑treated groups (Fig. 2C) (*P*<0.05).

**Fig. 3:**
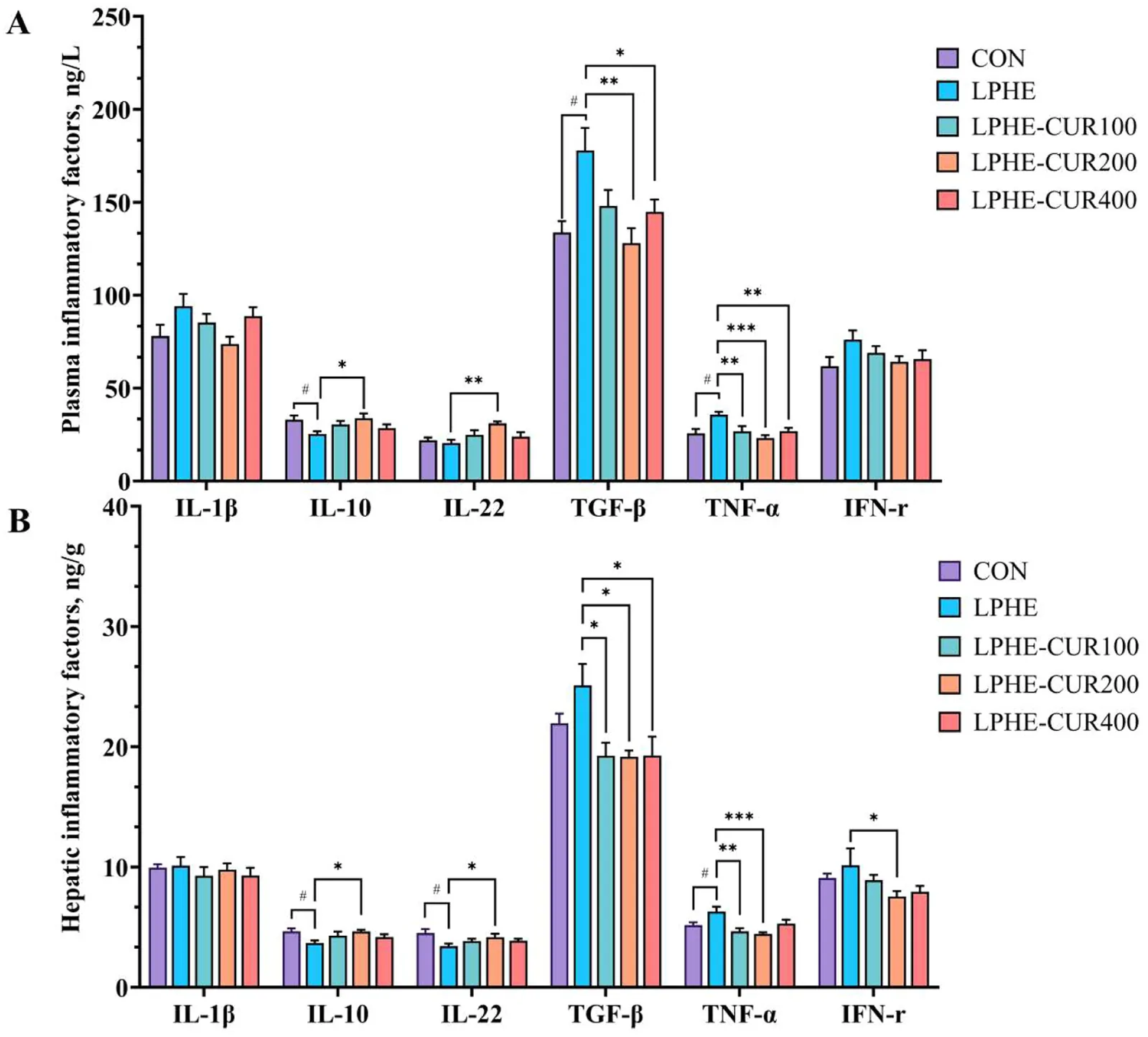
The effects of low protein with high energy diet supplemented with curcumin (CUR) on inflammatory factors secretion of plasma (A) and hepatic (B) in broiler breeder hens. CON, basal diet; LPHE, low protein high energy diet; LPHE_CUR100, low protein high energy diet supplemented with 100 mg/kg curcumin; LPHE_CUR200, low protein high energy diet supplemented with 200 mg/kg curcumin; LPHE_CUR400, low protein high energy diet supplemented with 400 mg/kg curcumin; Data are presented as mean value ± SD. # means the significant difference of LPHE compared to CON group, and # means P < 0.05. * means the significant difference of LPHE_CUR100, LPHE_CUR200, and LPHE_CUR200 compared to LPHE group, and * means P < 0.05, ** means P < 0.01, *** means P < 0.001.

Alterations in inflammatory cytokine levels among the experimental groups are illustrated in Fig. 3. Hens fed the LPHE diet exhibited higher plasma concentrations of TGF‑β and TNF‑α relative to the control group, along with elevated hepatic TNF‑α levels (*P* < 0.05). In contrast, anti‑inflammatory cytokines were suppressed in the LPHE group, as evidenced by reduced IL‑10 levels in plasma and decreased hepatic IL‑10 and IL‑22 concentrations (*P* < 0.05). Compared with the LPHE group, plasma concentrations of TGF‑β and TNF‑α were lower in the LPHE_CUR200 and LPHE_CUR400 groups. Moreover, hepatic TGF‑β and TNF‑α concentrations were reduced in the LPHE_CUR100 and LPHE_CUR200 groups (*P* < 0.05). However, the plasma and hepatic concentrations of IL-10 and IL-22 in LPHE_CUR200 group were significantly increased and the hepatic concentrations of IFN-γ in the LPHE-CUR200 group was significantly decreased (*P*<0.05) (Fig. 3).

### Hepatic metabolomic analysis

**Fig. 4:**
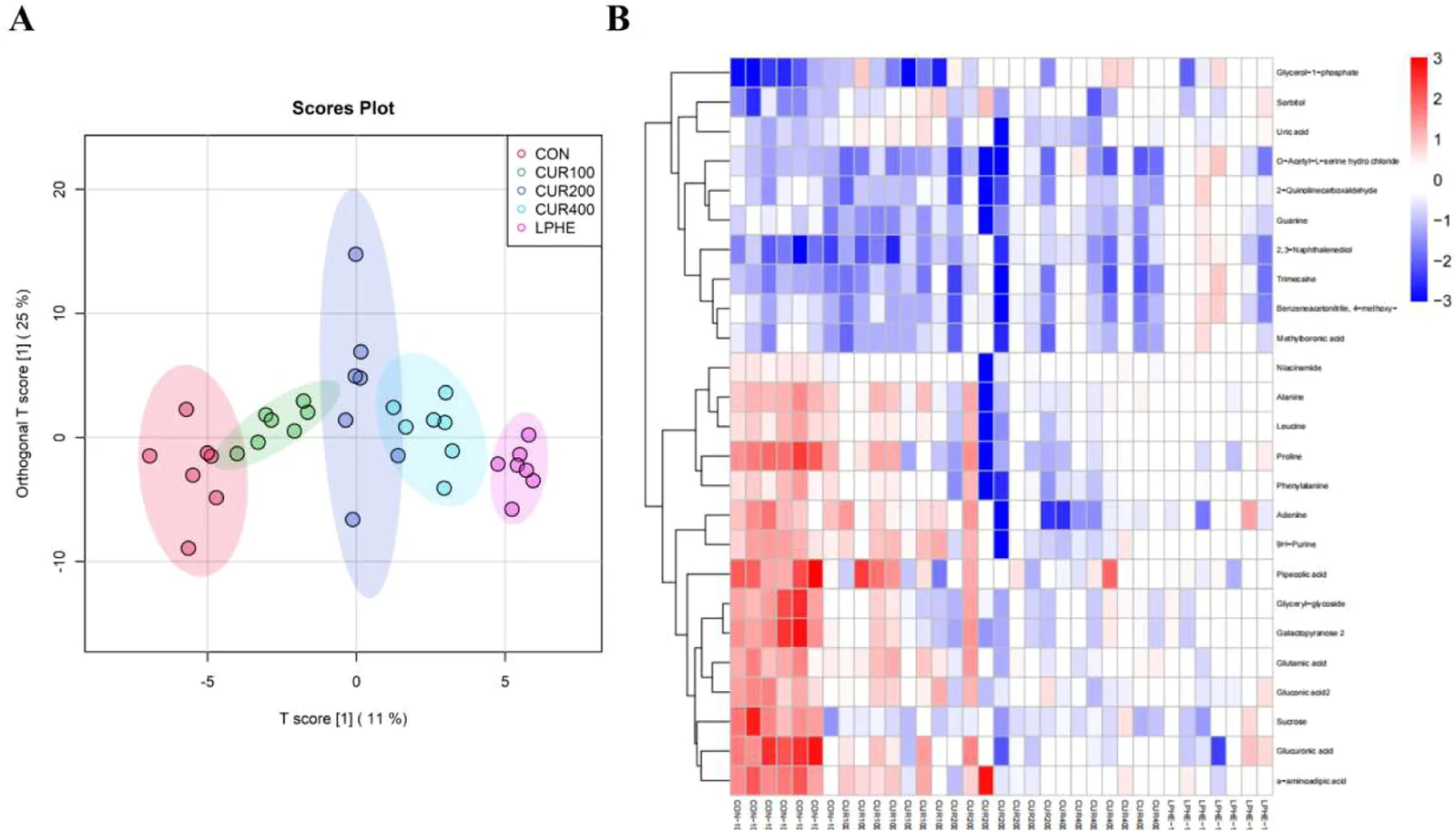
The effects of low protein with high energy diet supplemented with curcumin (CUR) on hepatic metabolites changes in broiler breeder hens. (A) the OPLS-DA plot; (B) The heatmap of differential metabolites exhibited similar patterns of variation. CON, basal diet; LPHE, low protein high energy diet; LPHE_CUR100, low protein high energy diet supplemented with 100 mg/kg curcumin; LPHE_CUR200, low protein high energy diet supplemented with 200 mg/kg curcumin; LPHE_CUR400, low protein high energy diet supplemented with 400 mg/kg curcumin.

The OPLS‑DA score plot presented in Fig. 4A demonstrates a distinct separation among the experimental groups based on hepatic metabolite profiles. Relative to the CON group, birds fed the LPHE diet showed the largest separation distance in the multivariate space, indicating pronounced alterations in liver metabolism. Conversely, the LPHE_CUR100 group clustered most closely with the control group, suggesting that CUR supplementation at this level resulted in the least metabolic deviation from normal hepatic profiles.

The heatmap of differential metabolites exhibited similar patterns of variation (Fig. 4B). The main differential metabolites among groups included glycerophosphate, uric acid, sorbitol, o-acetyl-L-serine hydro chloride, 2-quinolinecarboxaldehyde, guanine, 2,3-naphthalenediol, trimecaine, methyboronic acid, niacinamide, alanine, leucine, proline, phenylalanine, adenine, 9H-purine, pipecolic acid, glycer yl-glycoside, galaclopyranose 2, gluconic acid, glutamic acid, sucrose, glucuronic acid, a-aminoadipic acid.

**Fig. 5:**
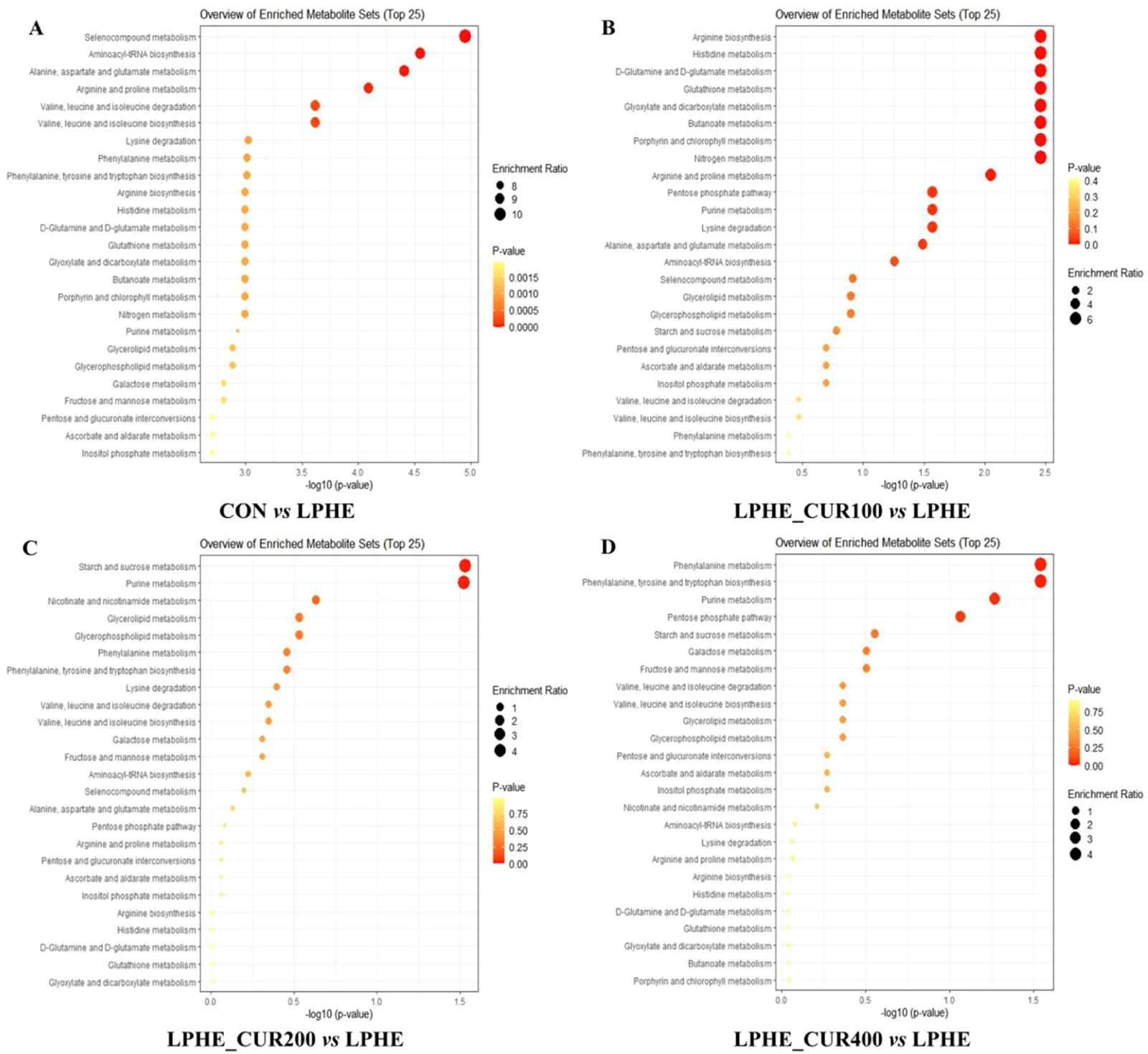
The effects of low protein with high energy diet supplemented with curcumin (CUR) on hepatic metabolites changes by KEGG pathway analysis in broiler breeder hens. (A) CON VS LPHE; (B) LPHE VS LPHE_CUR100; (C) LPHE VS LPHE_CUR200; (D) LPHE VS LPHE_CUR400; CON, basal diet; LPHE, low protein high energy diet; LPHE_CUR100, low protein high energy diet supplemented with 100 mg/kg curcumin; LPHE_CUR200, low protein high energy diet supplemented with 200 mg/kg curcumin; LPHE_CUR400, low protein high energy diet supplemented with 400 mg/kg curcumin.

As shown in Fig. 5, KEGG pathway analysis revealed that the CON group showed significant alterations primarily in metabolic pathways related to aminoacyl-tRNA biosynthesis, alanine, aspartate, and glutamate metabolism, arginine and proline metabolism, arginine biosynthesis, histidine metabolism, D-glutamine and D-glutamate metabolism, glutathione metabolism and glyoxylate and dicarboxylate metabolism, compared with the LPHE group (Fig. 5A). On the contrary, there were no significant differences in the following metabolic components, such as arginine biosynthesis, histidine metabolism, D-glutamine and D-glutamate metabolism, glutathione metabolism, glyoxylate and dicarboxylate metabolism, porphyrin and chlorophyll metabolism, glycerolipid metabolism and glycerophospholipid metabolism. However, a comparison between the LPHE and LPHE_CUR100 groups revealed that the significantly altered metabolic pathways were primarily enriched in arginine biosynthesis, histidine metabolism, D-glutamine and D-glutamate metabolism, glutathione metabolism, glyoxylate and dicarboxylate metabolism, porphyrin and chlorophyll metabolism, arginine and proline metabolism, aminoacyl-tRNA biosynthesis, glycerolipid metabolism and glycerophospholipid metabolism (Fig. 5B). The LPHE_CUR200 group showed significant alterations primarily in metabolic pathways related to starch and sucrose metabolism, purine metabolism, nicotinate and nicotinamide metabolism, glycerolipid metabolism, glycerophospholipid metabolism, phenylalanine metabolism and phenylalanine, tyrosine and tryptophan biosynthesis (Fig. 5C). The LPHE_CUR400 group showed significant alterations primarily in metabolic pathways related to phenylalanine metabolism, phenylalanine, tyrosine and tryptophan biosynthesis, purine metabolism, starch and sucrose metabolism, galactose metabolism, glycerolipid metabolism and glycerophospholipid metabolism (Fig. 5D).

### Hepatic bile acid profiles and related gene expression

**Fig. 6:**
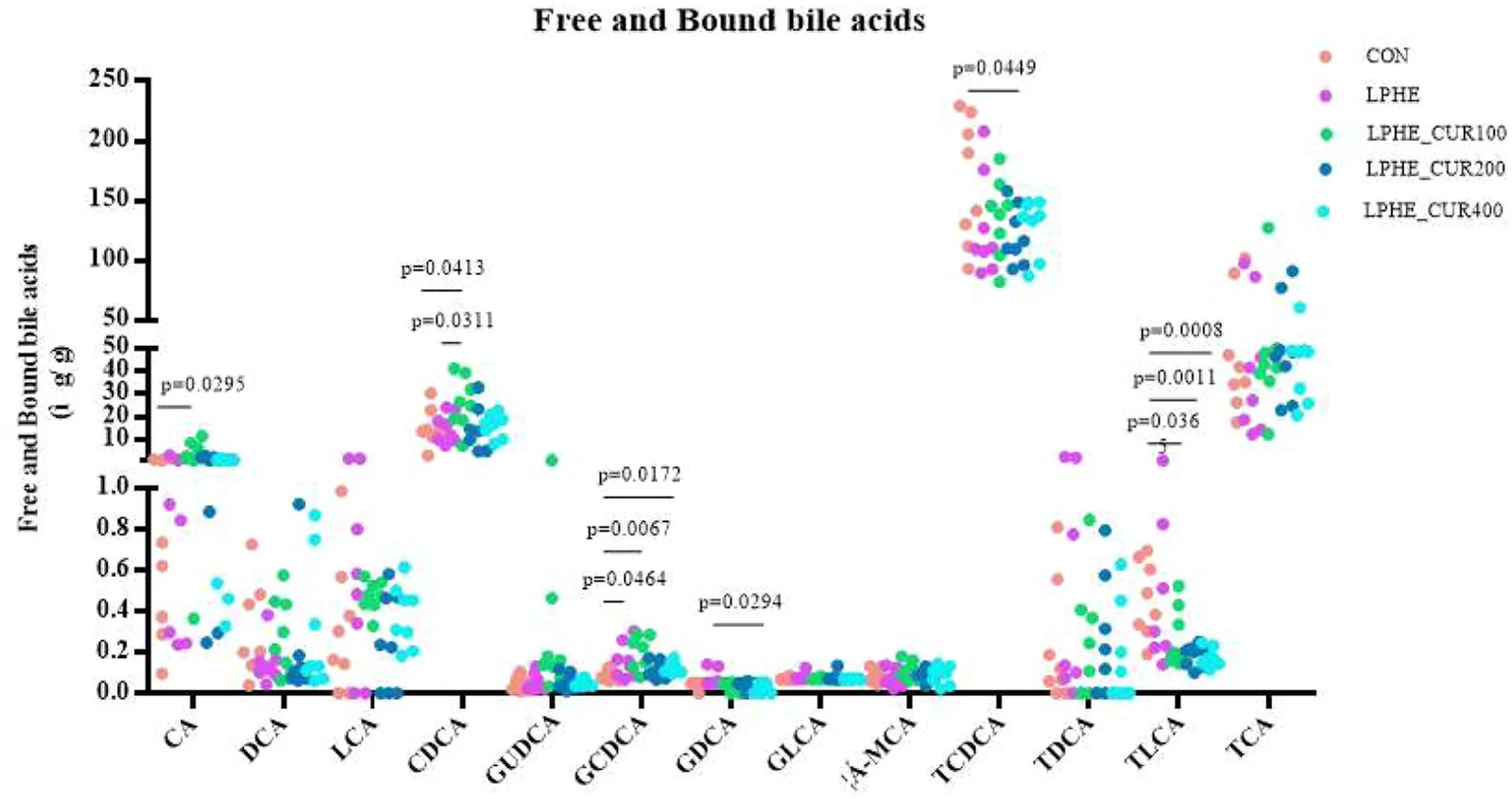
The effects of low protein with high energy diet supplemented with curcumin (CUR) on the hepatic concentrations of primary and secondary bile acids in broiler breeder hens. CON, basal diet; LPHE, low protein high energy diet; LPHE_CUR100, low protein high energy diet supplemented with 100 mg/kg curcumin; LPHE_CUR200, low protein high energy diet supplemented with 200 mg/kg curcumin; LPHE_CUR400, low protein high energy diet supplemented with 400 mg/kg curcumin; CA: cholic acid; DCA:deoxycholic acid; LCA: lithocholic acid; CDCA: chenodexycholic acid; GUDCA: Glycoursodeoxycholic acid; GCDCA: Glycoursodeoxycholic acid; GDCA: Glycodeoxycholic acid; GLCA: Glycolithocholic acid; TCDCA: Taurochenodeoxycholic acid; TDCA: Taurodeoxycholic acid; TLCA: Tauro lithocholic acid; TCA: Taurocholic acid; ‌α-MCA: α-Muricholic acid. Data are presented as mean value ± SD.

Changes in hepatic bile acid concentrations are illustrated in Fig. 6. Relative to the control group, hens receiving the LPHE diet exhibited lower levels of cholic acid (CA), deoxycholic acid (DCA), and chenodeoxycholic acid (CDCA), whereas glycochenodeoxycholic acid (GCDCA), glycodeoxycholic acid (GDCA), and taurolithocholic acid (TLCA) were present at higher concentrations (*P* < 0.05). In the LPHE_CUR100 group, hepatic CA and CDCA levels were elevated compared with those observed in the LPHE group (*P* < 0.05). A significant increase in DCA concentration was detected in the LPHE_CUR200 group (*P* < 0.05). In contrast, concentrations of GCDCA, GDCA, and TLCA were reduced in CUR‑treated groups when compared with the LPHE group (*P* < 0.05).

**Fig. 7:**
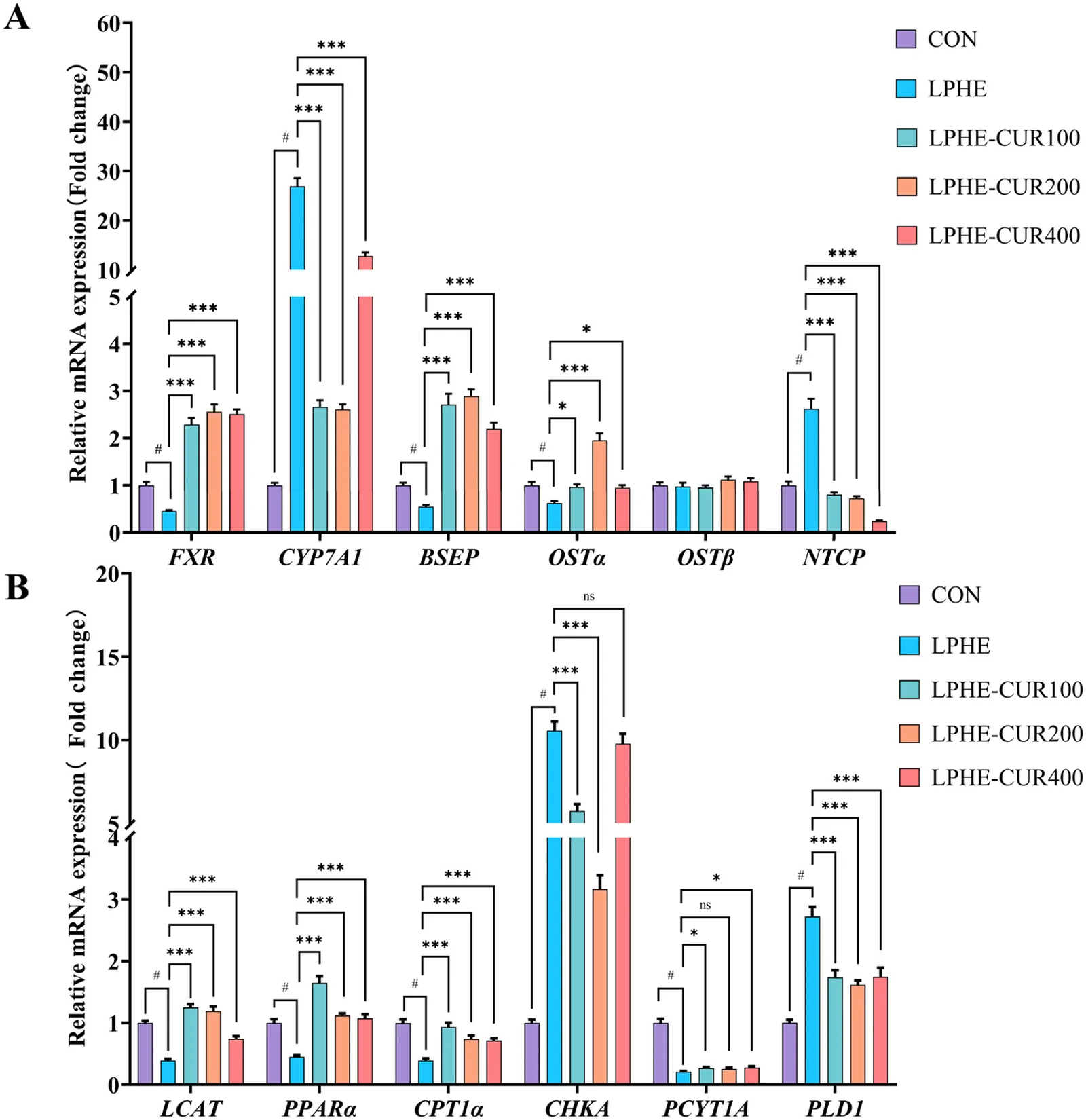
The effects of low protein with high energy diet supplemented with curcumin (CUR) on the hepatic mRNA expression of genes related to bile acids metabolism (A) and choline-metabolizing genes (B) in broiler breeder hens. CON, basal diet; LPHE, low protein high energy diet; LPHE_CUR100, low protein high energy diet supplemented with 100 mg/kg curcumin; LPHE_CUR200, low protein high energy diet supplemented with 200 mg/kg curcumin; LPHE_CUR400, low protein high energy diet supplemented with 400 mg/kg curcumin; Data are presented as mean value ± SD. # means the significant difference of LPHE compared to CON group, and # means P < 0.05. * means the significant difference of LPHE_CUR100, LPHE_CUR200, and LPHE_CUR200 compared to LPHE group, and * means P < 0.05, ** means P < 0.01, *** means P < 0.001.

As shown in Fig. 7, the mRNA expression of *FXR, BSEP, OSTα, PPARα, LCAT* and *CPT1α* in the LPHE group was significantly reduced (*P*<0.05), while the expression of these genes was significantly elevated in CUR-treated groups (*P*<0.05); the mRNA expression of *CYP7A1, NTCP, CHKA* and *PLD1* in the LPHE group was significantly increased (*P*<0.05), while their expression in the LPHE_CUR100 and LPHE_CUR200 groups was significantly decreased (*P*<0.05).

### Gut microbiome

Cecal microbial profiling revealed 1,036 operational taxonomic units (OTUs) across the 50 collected samples, with 771 OTUs were common among all groups.

**Fig. 8:**
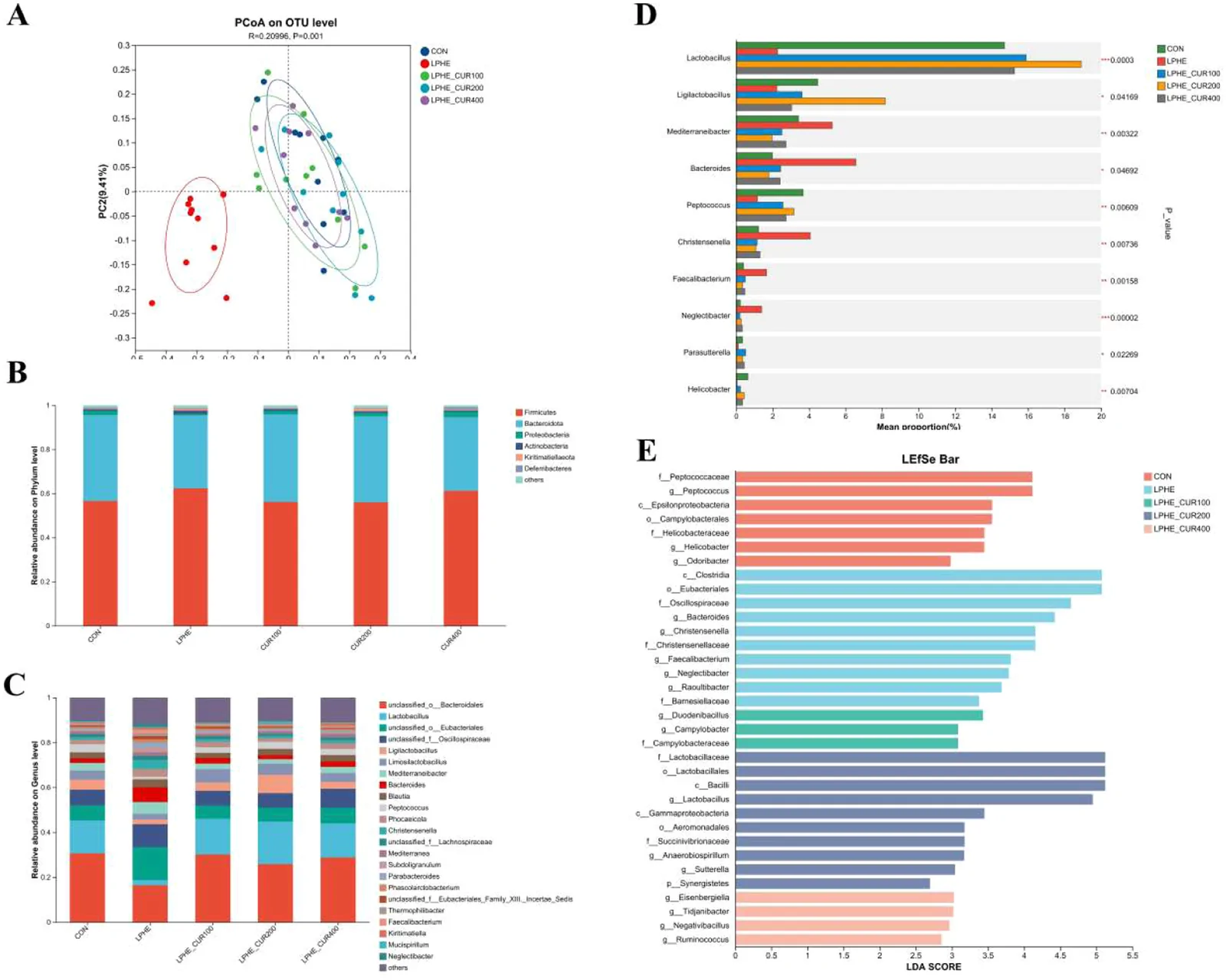
The effects of low protein with high energy diet supplemented with curcumin (CUR) on the β-diversity and composition of cecal microbiome in broiler breeder hens. (A) Plots of PCoA based on OUT level. (B) Microbial community composition of the cecum at the phylum level. (C) Microbial community composition of the cecum at the genus level. (D) Kruskal-Wallis H test of different species at the genus level. (E) LEfSe analysis of different species of cecal microbiota. CON, basal diet; LPHE, low protein high energy diet; LPHE_CUR100, low protein high energy diet supplemented with 100 mg/kg curcumin; LPHE_CUR200, low protein high energy diet supplemented with 200 mg/kg curcumin; LPHE_CUR400, low protein high energy diet supplemented with 400 mg/kg curcumin.

Assessment of microbial diversity (Table S3) demonstrated that CUR supplementation was associated with pronounced alterations in alpha diversity. CUR-treated groups exhibited higher richness and coverage indices (observed species, Chao, ACE; *P*<0.05) than the LPHE group, while Shannon and Simpson indices showed no significant differences across all groups (*P*>0.05). As shown in Fig. 8A, the scatter plot of PCoA highlighted obvious differences in the microbiota of the cecum among the groups (*P*=0.001). Substantial changes in cecal microbial structure were observed between the CON and LPHE groups, whereas samples from all CUR-treated groups clustered closely with the CON group.

The relative abundances of the five predominant bacterial phyla-Firmicutes, Bacteroidota, Proteobacteria, Actinobacteria, and other minor phyla—are presented in Fig. 8B. At the phylum level, compared with the LPHE group, a lower relative abundance of Firmicutes was detected in breeder hens receiving CUR at 100 or 200 mg/kg (*P* < 0.05). At the genus level (Fig. 8C), the LPHE diet was associated with higher abundances of *unclassified Eubacteriales, unclassified Oscillospiraceae, Bacteroides, Phocaeicola*, and *Christensenella*, while reducing the abundances of *unclassified Bacteroidales, Lactobacillus, Ligilactobacillus, Limosilactobacillus*, and *Peptococcus* (*P* < 0.05). These LPHE‑associated shifts in microbial composition were largely attenuated in the CUR‑supplemented groups.

Consistent with these observations, Kruskal–Wallis analysis (Fig. 8D) showed that the LPHE group exhibited significantly reduced abundances of *Lactobacillus, Ligilactobacillus*, and *Peptococcus*, along with increased levels of *Mediterraneibacter, Bacteroides, Christensenella, Faecalibacterium*, and *Neglectibacter* (*P* < 0.05). In contrast, these genus‑level changes were reversed in hens supplemented with CUR at 100, 200, or 400 mg/kg (*P* < 0.05). Further characterization using LEfSe analysis (LDA score > 5.0; Fig. 8E) revealed clear compositional differences in the cecal microbial community among treatment groups. The LPHE diet was characterized by enrichment of Clostridia (class) and Eubacteriales (order), whereas CUR supplementation at 200 mg/kg was associated with increased representation of Lactobacillales (order), Lactobacillaceae (family), and Bacilli (class).

### Correlation analyses between bile acids, inflammatory cytokines, lipids, and microbial communities

**Fig. 9:**
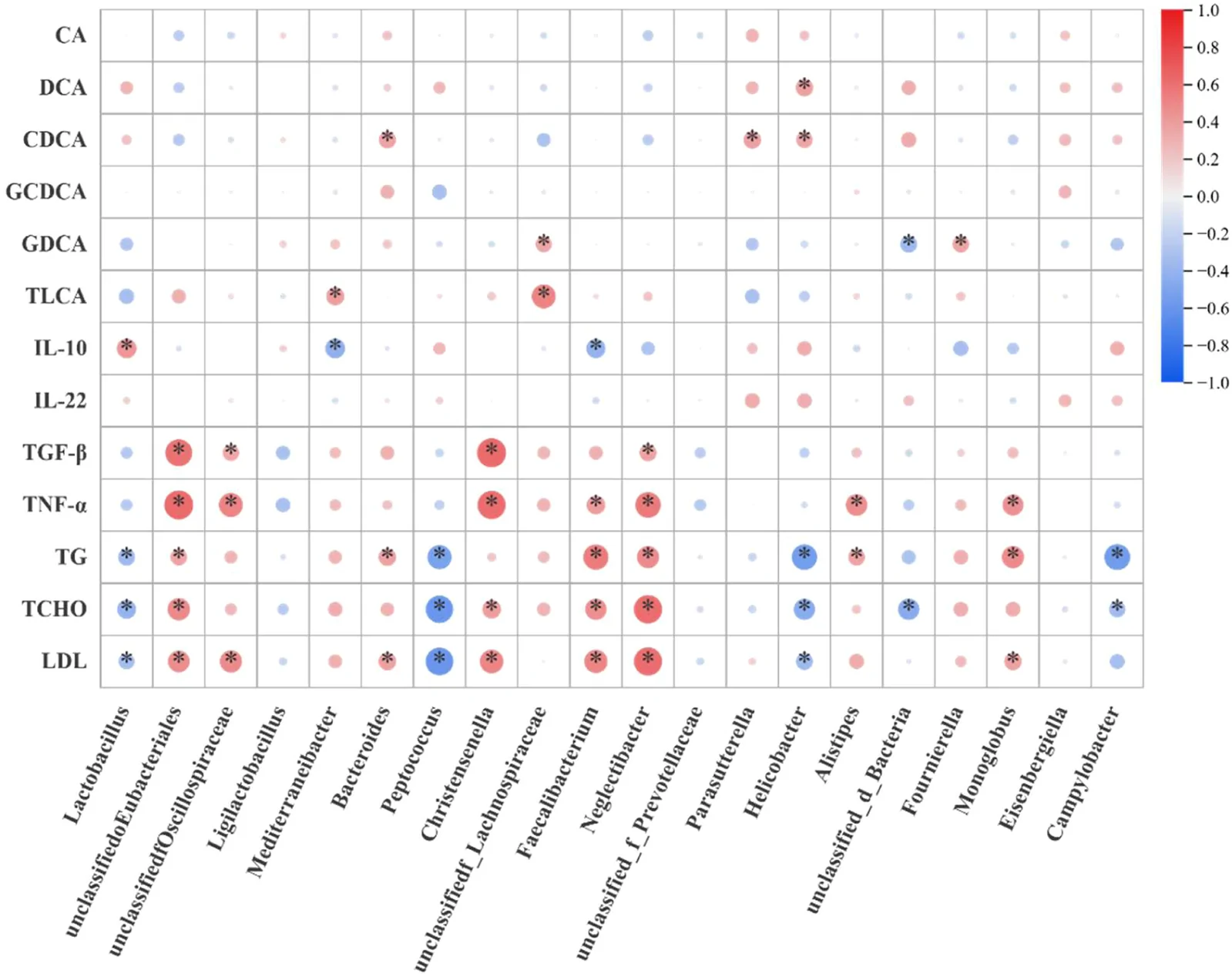
Heatmap of spearman correlation analysis between the genus level of gut microbiota and liver lipids, inflammatory cytokines and bile acids. Brown shading with P < 0.05 represents a significant negative correlation, green shading with P < 0.05 represents a significant positive correlation, white shading represents no correlation, n = 12. * P < 0.05; ** P < 0.01; *** P < 0.001.

Correlation analysis revealed that hepatic TG and TCHO levels were inversely related to the relative abundances of *Lactobacillus, Peptococcus*, and *Helicobacter*, while exhibiting positive relationships with *Faecalibacterium* and *Neglectibacter* (*P* < 0.01) (Fig. 9).

In addition, CDCA concentrations showed positive associations with the abundances of *Bacteroides* and *Helicobacter* (*P* < 0.05). Analysis of inflammatory factors further indicated that TNF‑α levels were positively linked to Eubacteriales, *Christensenella*, and *Neglectibacter* (*P* < 0.05). Similarly, TGF‑β concentrations demonstrated positive associations with Eubacteriales and *Christensenella* (*P* < 0.05).

## Discussion

Prior research has shown that FLS has a negative impact on the egg-laying performance in hen enterprises, leading to financial disadvantages for the hen farming industry. The results revealed a significant reduction in productive performance among *Qingyuan* partridge broiler breeder hens fed the LPHE diet. These findings are consistent with earlier studies on White Leghorn laying hens (Rozenboim et al., 2016), Jing Fen (Zhang et al., 2024), and Lohmann laying hens (Li et al., 2025) fed with LPHE diet. The LPHE diet increased abdominal fat, similar to observations in 56-week-old Jing Fen hens (Zhang et al., 2024). Data analysis indicated that adding CUR for 8 weeks reduced the hepatic and abdominal metrics and improved egg production in broiler breeder hens. A study focusing on Japanese quails concluded that adding CUR significantly improved hepatic functionality reflected by ALT and AST levels of blood (Reda et al., 2020). Accordingly, H&E and Oil Red O staining revealed a significant decrease in hepatic lipid droplets in the CUR-treated group. What’s more, CUR also enhanced anti-inflammatory function through the reduction of colonic inflammatory cytokines, including TNF-α and IL-1β, in male Balb/c mice (Jiang et al., 2025). CUR markedly suppressed hepatic TNF-α expression in postnatal rats under ketamine anesthesia (Afshar Ghahremani et al., 2024) and the high-fat diet/streptozotocin-induced type 2 diabetic mice (Li et al., 2025), but had no effect on cecal TNF-α, INF-γ, IL-1β, and TGF-β in another study (Chen et al., 2024). Crucially, IL-22, as a cytokine with many immunoregulatory effects, exerts opposite influences on hepatic lipid accumulation under a high-fat diet (Nogues et al., 2025). Our findings indicated that CUR decreased plasma and hepatic concentrations of TNF-α and TGF-β, while increasing plasma concentrations of IL-22 and IL-10, highlighting its potential role in modulating inflammatory responses. Collectively, CUR may improve metabolism, liver health, and laying performance in broiler breeder hens. Based on UPLC-MS/MS metabolomics analysis, we have also demonstrated that CUR exerts a dual impact on both protein and hepatic lipid metabolism. Especially, changes in arginine metabolism are often closely related to inflammatory response or immune regulation in fatty liver disease associated with metabolic dysfunction (Mo et al., 2026). The enrichment of glycerolipid and glycerophospholipid metabolism reflects alterations in cell membrane remodeling and energy mobilization (Xu et al., 2026), which is closely linked to hepatic steatosis (Luo et al., 2026). These observations strengthen the possibility that oxidative stress and inflammatory damage may be alleviated by this coordinated metabolic reprogramming.

BAs are increasingly recognized as pivotal signaling molecules in regulating lipid, energy metabolism, gut microbiota, and immunity by activating enterohepatic receptors (Ruan et al., 2022; Fuchs et al., 2025). In the literature, FXR activated by CDCA, CA and DCA in the liver and also modulated their synthesis, transport and excretion (Wahlstrom et al., 2016). Additionally, FXR activation in hepatic macrophages suppresses IL-1β and TNF-α production, inhibits hepatic lipogenesis, and promotes peripheral triglyceride clearance (Huang et al., 2025), while CUR improved nonalcoholic simple fatty liver disease in humans by altering BAs (He et al., 2024). Our observations suggested that CUR treatment may increase the levels of DCA, CA, and CDCA in liver. Correspondingly, DCA limits the secretion of TNF-α, IFN-γ, IL-6 and IL-1β in murine colonic macrophages, thereby facilitating their differentiation into an IL-10-producing phenotype (Chen et al., 2019). Actually, CDCA and its derivatives have been extensively evaluated as FXR agonists for treating NAFLD (Gottlieb et al., 2019). Notably, CDCA generated from the alternative BA pathway enhances hepatic FXR activation and facilitates BA excretion, whereas TCDCA acts as a natural FXR antagonist (Li et al., 2022). This study found that CUR also decreased hepatic conjugated BAs, including GCDCA, TCDCA, TLCA, and GDCA. In accordance, conjugated bile acids play a pivotal role in modulating adiposity, energy metabolism, and glucose homeostasis (Zheng et al., 2021). Meanwhile, GCDCA exhibits immunosuppressive effects (Lin et al., 2026), whereas TLCA is a pro-inflammatory BA and its elevation promotes bile ecological imbalance and cancer (Liwinski et al., 2020). Collectively, our findings indicate that CUR may improve the immune status of fatty liver broiler chickens by altering the BAs pool and regulating key hepatic genes involved in bile acid metabolism (Fuchs et al., 2025). Dysregulated phosphatidylcholine metabolism and its key synthetic genes is a core mechanism of hepatic lipid deposition. In the present study, CUR improved lipid metabolism by upregulating *LCAT, CPT1* and *PPARα* to promote reverse lipid transport (Janilkarn-Urena et al., 2023), while also potentially balancing glycerophospholipid synthesis and degradation by regulating *CHKA* and *PLD1* expression through modulation of hepatic phospholipase activity (Liu et al., 2025). These findings provide a genetic basis for alleviating lipid metabolic disturbances in LPHE broilers, aligning with the plasma and hepatic lipid metabolism biomarkers documented in this study. Moreover, whether CUR's regulatory effects on bile acid metabolism-related signaling pathways are dependent on these receptors needs to be further elucidated.

Host health may be supported by a diverse microbial community within the gastrointestinal system. Understanding CUR's biological activity hinges on its bidirectional interplay with gut microbiota. As previously stated, CUR might exert its effects at the intestinal level through influencing the makeup and microbial diversity of intestinal flora, which may contribute to improve liver inflammation and modulate signaling pathways (He et al., 2024). In the current experiment, CUR supplementation to LPHE feed could effectively reverse the decrease in gut microbiota richness and declining trends in the abundance of *Lactobacillus* and *Ligilactobacillus*. Lactobacillus and Ligilactobacillus improve NAFLD via gut microbiota modulation of lipid metabolism and anti-inflammatory effects, with their derived enzymes mediating BA uncoupling (Sun et al., 2019; Yu et al., 2021; Park et al., 2025; Wang et al., 2025). Our research indicated that TG, TCHO, and LDL negatively correlated with Lactobacillus abundance, whereas IL-10 correlated positively. Consistent with the study’s results, *Lactobacillus* has been shown to convert primary BAs into secondary BAs, including DCA (Li et al., 2025). As a beneficial bacteria, *Peptococcus* is positively correlated with anti-inflammatory function and body weight of broilers (Jiang et al., 2024). Notably, the abundance of *Peptococcus* also showed significant increase after CUR supplementation in LPHE broliers. Conversely, we observed a marked reduction in several key gut bacterial families, notably *Mediterraneibacter* and *Christensenella. Mediterraneibacter* species are capable of ethanol production, and their overabundance may positively correlate with liver adiposity (Xiao et al., 2025). In addition, *Christensenella* showed a notable association with obesity-related parameters, as well as obesity-related metabolic disorders, in the mice (Song et al., 2021). Our results also revealed a negative correlation between TG and TCHO levels and *Peptococcus* abundance, while a positive correlation was observed with *Christensenella* abundance. Moreover, a positive correlation was identified between the levels of TNF-α and TGF-β and *Christensenella*, whereas a negative correlation was detected between IL-10 and *Mediterraneibacter*. In line with our observations, CUR at a dosage of 200 mg/kg demonstrated a more pronounced therapeutic effect than 100 mg/kg in type 2 diabetic rats (Shamsi-Goushki et al., 2020). It is plausible that the effect of CUR on the body is influenced by both the dosage level and individual metabolic capacity, which may in turn be affected by the composition of the intestinal flora. These alterations, coupled with the elevation of unconjugated bile acids in hepatic tissue, substantiate that a dosage of 100∼200 mg/kg CUR facilitates the deconjugation of bile acids. Mechanistically, CUR administration facilitated the expansion of the genera *Lactobacillus* and *Ligilactobacillus*, which may in turn enhance the deconjugation of bile acids, thereby upregulating hepatic FXR expression and alleviating liver lipid deposition. Therefore, further studies could be conducted to identify the key metabolites that regulate the intestinal microbiota and lipid metabolism..

## Conclusions

In summary, dietary supplementation with 100 to 200 mg/kg CUR could not only reduce LPHE induced liver lipid metabolism disorders in broiler breeder hens, but also improve egg production performance by reducing liver inflammation, optimizing bile acid homeostasis and intestinal microbiota structure, which may be associated with its modulation of the gut microbiota- conjugated BAs-FXR signaling pathway. Wang et al., 2025; Magdy Wasfy et al., 2025; Liang et al., 2025; Shini et al., 2020

## Funding

This study was supported by International Network for Agricultural Science and Technology Cooperation (GHZX2025-XZ05), China Agriculture Research System of MOF and MARA (CARS-41-G06 and CARS-40-G06), 10.13039/501100001809National Natural Science Foundation of China (32402816), 10.13039/501100003453Guangdong Natural Science Found (2024A1515012121), Guangdong S&T Program (2026B0202150004), Guangdong Feed Industry Technology System (2026CXTD14), the Independent Research Fund of State Key Laboratory of Swine and Poultry Breeding Industry (ZQQZ-G13, ZQQZ-K03, GDNKY-ZQQZ-K23, 2023QZ-NK11), Special Funding for the Construction of the High-Level Academy of Agricultural Sciences (NYQS202614). Supporting Program for the Research of State Key Laboratory of Swine and Poultry Breeding Industry, Key Laboratory of Animal Nutrition and Feed Science in South China, Ministry of Agriculture and Rural Affairs, Guangdong Provincial Key Laboratory of Animal Breeding and Nutrition.

## CRediT authorship contribution statement

**Jinling Ye**: Writing-original draft, Visualization, Methodology, Investigation, Data curation, Formal analysis. **Yujie Huang**: Methodology, Data curation, Validation, Software. **Ahmed M. Fouad**: Methodology, Data curation, Writing-review & editing. **Sai Zhang**: Methodology, Data curation, Validation, Software. **HebatAllah K. El-Senousey**: Methodology, Data curation, Validation, Software. **Zhongyong Gou**: Methodology, Data curation, Validation, Software. **Chang Zhang**: Methodology, Data curation, Validation, Software. **Zongyong Jiang**: Supervision, Methodology, Resources. **Shouqun Jiang**: Supervision, Methodology, Funding acquisition. **Dong Ruan**: Writing-review & editing, Supervision, Methodology, Funding acquisition, Conceptualization.

## Disclosures

The authors declare that they have no competing financial interests or personal relationships that may have influenced the work reported in this study.

## Data Availability

The datasets presented in this study are available from the corresponding author upon reasonable request.

## Footnotes

Supplementary material associated with this article can be found, in the online version, at doi:10.1016/j.psj.2026.107650.
