# Supplementary material for: Curcumin ameliorates fatty liver hemorrhagic syndrome in broiler breeders by regulating lipid metabolism via the gut-liver axis
Source: Poult Sci. 2026 Aug 18;105(12):107650. doi: 10.1016/j.psj.2026.107650 (PMC13594817; doi:10.1016/j.psj.2026.107650)
Supplement: Supplementary file 1 [file mmc1.docx]

**Table S1** Ingredients and nutritional composition of the basal diet and LPHE diet for broiler breeders (as-fed basis) %

| Items | Control diet (CON) | Low protein and high-metabolic-energy diet (LPHE) |
| --- | --- | --- |
| Corn | 63.74 | 66.57 |
| Soybean meal | 25.20 | 18.80 |
| Corn gluten meal | 1.50 | 0.00 |
| Plam oil | 0.00 | 4.50 |
| *L-*Lys.HCl (98.5%) | 0.00 | 0.18 |
| *DL*-methionine (99%) | 0.13 | 0.18 |
| *L*-threonine (99%) | 0.03 | 0.06 |
| L- isoleucine (98.5%) | 0.00 | 0.06 |
| L-Arginine (98%) | 0.00 | 0.20 |
| Limestone | 6.80 | 6.80 |
| CaHPO_4_ | 1.30 | 1.35 |
| NaCl | 0.30 | 0.30 |
| Premix^1^ | 1.00 | 1.00 |
| Total | 100.00 | 100.00 |
| Nutrient level^2^ |  |  |
| Metabolizable energy, kcal/kg | 2655 | 2955 |
| Crude protein | 17.00 | 13.50 |
| Calcium | 3.00 | 3.00 |
| Total phosphorus | 0.60 | 0.58 |
| Non-phytate phosphorus | 0.36 | 0.36 |
| Total Lys | 0.80 | 0.80 |
| Total Met | 0.40 | 0.40 |
| Total Met+Cys | 0.68 | 0.68 |
| Total Thr | 0.61 | 0.61 |
| Total Trp | 0.17 | 0.17 |
| Total Ile | 0.56 | 0.56 |
| Total Arg | 1.03 | 1.03 |

^1^The premix provided the following per kg of the diets： vitamin A, 12,000 IU; vitamin D_3_, 3,000 IU; vitamin E, 30 mg; vitamin K_3_, 0.2 mg; vitamin B_1_, 3.0 mg; vitamin B_2_, 9.0 mg; vitamin B_6_, 3.0 mg; vitamin B_12_, 0.01 mg; choline, 1,050 mg; nicotinic acid, 25 mg; pantothenic acid, 12 mg; folic acid, 1.5 mg; biotin, 0.15 mg; Fe, 80 mg; Cu, 8.0 mg; Mn, 90 mg; Zn, 80 mg; I, 0.40 mg; Se, 0.12 mg.

**Table S2** Primer sequences used for real-time quantitative PCR.

| Genes | Direction | Primer sequence (5′-3′) | NCBI references |
| --- | --- | --- | --- |
| *FXR* | Forward  Reverse | CAGAAAGAATGCAGCGGCTC  CAAACTGCCCCATTTTGCGA | NM_204113.2 |
| *CYP7A1* | Forward  Reverse | TGACCCAGCAGAAGGAAACA  ACCCAGGTGTTAGGCTGAAA | NM_001001753.1 |
| *BSEP* | Forward  Reverse | TGTTGGTGCTAGTGGAGCTG  TGCTGTGGCAAGTCCATGAT | XM_040676679.2 |
| *OSTα* | Forward  Reverse | GAAACCAAGGCAGTCAGT  ATCATCTGCCAGCTCCAT | NM_001277697.1 |
| *OSTβ* | Forward  Reverse | GAGGAGAAAGCAGCACAA  CCAGCACAAGGACATCAT | XM_046899178.1 |
| *NTCP* | Forward  Reverse | AGACAGGGATGGTTGTGCTT  CTGAGGGGAGATGGTGATGT | XM_015287931 |
| *PPARα* | Forward  Reverse | ACGAATGCCAAGGTCTGAGA  TGCAAGGATGACTCTGGCTT | NM_001001464.1 |
| *LCAT* | Forward  Reverse | TGTGGGAGGACATGAAGGAC  TCTGAGCTGCGTTTGTTGAC | NM_001293094.2 |
| *CPT1α* | Forward  Reverse | GGCTCTGGCAGGAGCTACA  AGTAGGTCAGGACACGCTCA | XM_040700878.1 |
| *CHKA* | Forward  Reverse | CATCAGCCCCATCAGAGGTG  TTCTGCCCCTTGCAAGTCAT | XM_040673174.2 |
| *PCYT1A* | Forward  Reverse | CATTCGGACTGAGGGAGCC  TGACAGGGCGATCCAAAGG | XM_025153429.3 |
| *PLD1* | Forward  Reverse | GCATCCCCTTCTCTGCCATC  GAGGCTTGGGACCTTCTTGG | XM_040679552.2 |
| *β-actin* | Forward  Reverse | GAGAAATTGTGCGTGACATCA  CCTGAACCTCTCATTGCCA | NM_205518 |

# FXR=Farnesoid X receptor; FGF19=fibroblast growth factor 19; CYP7A1=cytochrome P450 family 7 subfamily A member 1; MRP2= Multi-drug resistance protein 2; OSTα*=*organic solute transporter alpha; OSTβ*=*organic solute transporter beta; NTCP=Sodium taurocholate cotransporting polypeptide; PPARα*=*peroxisome proliferator activated receptor alpha; LCAT=lecithin-cholesterol acyltransferase; CPT1A=carnitine palmitoyltransferase 1A; CHKA=choline kinase alpha; PCYT1A=phosphate cytidylyltransferase 1 alpha; PLD1=phospholipase D1; PTDSS1=phosphatidylserine synthase 1; LPCAT2=lysophosphatidylcholine acyltransferase 2.

**Table S3** Effects of CUR on *α*-diversity of broiler breeder hens fed with LPHE diet

| Item | Control | LPHE | LPHE-CUR100 | LPHE- CUR 200 | LPHE- CUR 400 | SEM | *P* Value |
| --- | --- | --- | --- | --- | --- | --- | --- |
| Species observed | 556.60^A^ | 459.10^Bb^ | 569.20^a^ | 569.60^a^ | 544.70^a^ | 18.404 | 0.002 |
| Shannon | 4.438 | 4.504 | 4.527 | 4.416 | 4.296 | 0.107 | 0.667 |
| Simpson | 0.041 | 0.038 | 0.043 | 0.048 | 0.048 | 0.007 | 0.217 |
| Chao | 685.12^A^ | 572.50^Bb^ | 693.64^a^ | 701.20^a^ | 675.59^a^ | 21.741 | 0.002 |
| Ace | 687.00^A^ | 566.53^Bb^ | 691.82^a^ | 705.02^a^ | 676.70^a^ | 20.258 | <0.001 |
| Goods＿coverage index/% | 98.88^B^ | 99.10^Aa^ | 98.84^b^ | 98.90^b^ | 98.87^b^ | 0.025 | <0.001 |

Mean values within a row with no common superscript differ significantly (*P*<0.05). CON, broiler breeder hens were fed the basal diet; LPHE, broiler breeder hens were fed low protein and high energy diet; LPHE_CUR100, broiler breeder hens were fed low protein and high energy diet supplemented with 100 mg/kg curcumin; LPHE_CUR200, broiler breeder hens were fed low protein and high energy diet supplemented with 200 mg/kg curcumin; LPHE_CUR400, broiler breeder hens were fed low protein and high energy diet supplemented with 400 mg/kg curcumin. ^a^Capital letters indicate statistically significant (*P*<0.05) differences between control group and LPHE group by Student’s t-test; small letters indicate statistically significant (*P*<0.05) differences of four groups (LPHE, LPHE-CUR100, LPHE-CUR200, LPHE-CUR400). Mean values within a row with no common superscript differ significantly (*P*<0.05). ^b^The *P*-value are representing the ANOVA analysis of four groups (LPHE, LPHE-CUR100, LPHE-CUR200, LPHE-CUR400) .
